# Label-free image-encoded microfluidic cell sorter with a scanning Bessel beam

**DOI:** 10.1063/5.0051354

**Published:** 2021-07-02

**Authors:** Xinyu Chen, Lauren Waller, Jiajie Chen, Rui Tang, Zunming Zhang, Ivan Gagne, Bien Gutierrez, Sung Hwan Cho, Chi-Yang Tseng, Ian Y. Lian, Yu-Hwa Lo

**Affiliations:** 1 Department of Electrical and Computer Engineering, University of California, San Diego, La Jolla, California 92093, USA; 2 Department of Bioengineering, University of California, San Diego, La Jolla, California 92093, USA; 3 College of Physics and Optoelectronics Engineering, Shenzhen University, Shenzhen 518060, China; 4 NanoCellect Biomedical, Inc., San Diego, California 92121, USA; 5 Department of Materials Science and Engineering, University of California, San Diego, La Jolla, California 92093, USA; 6 Department of Biology, Lamar University, Beaumont, Texas 77710, USA

## Abstract

The microfluidic-based, label-free image-guided cell sorter offers a low-cost, high information content, and disposable solution that overcomes many limitations in conventional cell sorters. However, flow confinement for most microfluidic devices is generally only one-dimensional using sheath flow. As a result, the equilibrium distribution of cells spreads beyond the focal plane of commonly used Gaussian laser excitation beams, resulting in a large number of blurred images that hinder subsequent cell sorting based on cell image features. To address this issue, we present a Bessel–Gaussian beam image-guided cell sorter with an ultra-long depth of focus, enabling focused images of >85% of passing cells. This system features label-free sorting capabilities based on features extracted from the output temporal waveform of a photomultiplier tube (PMT) detector. For the sorting of polystyrene beads, SKNO1 leukemia cells, and *Scenedesmus* green algae, our results indicate a sorting purity of 97%, 97%, and 98%, respectively, showing that the temporal waveforms from the PMT outputs have strong correlations with cell image features. These correlations are also confirmed by off-line reconstructed cell images from a temporal–spatial transformation algorithm tailored to the scanning Bessel–Gaussian beam.

## I. INTRODUCTION

Characterization, classification, and isolation of cell types among a heterogeneous population based on their stain-free morphological characteristics can yield significant biological insights, especially when coupled with phenotype–genotype correlations. Cell classification processes often require both the multiparametric spatial information of intracellular structures and high data volume analysis. In recent years, genome sequencing and population genomic analysis have had a profound impact in biological research by enabling high-volume comparative analysis, enabling new cell type discovery, and uncovering previously unknown cellular heterogeneities.^1^ This has significantly increased the need for methods capable of isolating cells of interest in a label-free environment to simplify the process flow, reduce cost, minimize cell disruptions by labeling, and overcome limitations of biomarker availability and specificity. Conventional methods of cell sorting include optical microscopy,^2^ deterministic lateral displacement,^3^ density gradient methods,^4^ and fluorescence and magnetic-activated cell sorting (FACS/MACS).^5–8^ However, these techniques suffer from some of the following aspects, including lack of specificity, low throughput, high cell loss, population-based sorting without single cell resolution, and the need for biochemical labeling.

A significant development in the field of label-free cell sorting is in the invention of an imaging flow cytometer/cell sorter. This microfluidic-based technology enables the highly informative morphological and spatial characterization of intracellular structures and subsequent sorting of cells of interest at a throughput of over 200 cells/s.^9^ Various possible configurations exist, each with unique characteristics and applications ranging from inexpensive, custom laboratory tools to precise clinical instruments. Examples of compatible on-chip cell sorting techniques include surface acoustic waves (SAWs),^10^ magnetic forces,^11^ and dielectrophoretic forces.^12^ Machine learning,^9^ artificial intelligence,^13^ and coupling with downstream microarray based systems^14^ are a natural progression of the field and have been applied.

Previously, our group developed an image-guided cell sorter using a fast scanning laser as the excitation source.^15^ In a simple microfluidic device suitable for low-cost, disposable applications that minimizes cross contamination,^16^ one-dimensional flow focusing confines the procession of cells into the center of the microfluidic channel only in one axis perpendicular to the flow direction. In the other perpendicular axis, however, the cell positions are not confined. As a result, particles in the flow channel tend to have a wide distribution in their positions affected by their size, stiffness, shape, and morphology. To extract image related features of high fidelity, keeping the cells at the focal spot of the interrogating beam is essential. Cells positioned outside the focal depth of the interrogating beam will give rise to blurred images. Furthermore, given the typical 10–15 *μ*m cell size, even for the cells located in the focal plane, a significant portion of the cell features can be out of focus. As a result, today’s image-guided flow cytometer cell sorters using a tightly focused Gaussian beam from a high numerical aperture (NA) objective face two major challenges: (a) to keep cells of different properties in the flow channel all in focus and (b) to keep all parts of the cells across their thickness along the optical axis in focus. Inability to meet the former requirement gives rise to a large number of out-of-focus cells, resulting in low throughput and biased analysis since some cell subpopulations tend to be in focus more than others. Failure to meet the latter requirement increases the risk of misleading the gating criteria for sorting since the apparent crisp cell image represents only the feature of one cross section of the cell, leaving features outside the focal plane blurry or not detectable. In this paper, we demonstrate a scanning Bessel beam system with extended focal depth to overcome the above limits and develop innovative approaches to perform image-guided cell sorting in a disposable microfluidic cartridge. The sorting criteria were directly determined from the image-encoded temporal waveform without image restoration. The system is simple to set up and can operate in a label-free manner.

Although not used in a flow cytometer system before, Bessel beam-based illumination microscopy methods have previously been leveraged to increase the depth of focus in biological specimens with near-isotropic spatial resolution, achieving significant merit in light-sheet microscopy, illumination microscopy, and electron microscopy.^17–19^ A Bessel beam is a diffraction-free mode solution of the Helmholtz equation and possesses a number of unique properties, which make it useful for imaging applications, including non-diffractive behavior and the ability to self-heal when partially obstructed.^20^ A mathematically ideal Bessel beam cannot exist as it is unbounded and carries an infinite amount of energy. An experimentally achievable approximation is to modulate the Bessel beam by a broad width Gaussian function, which is called a Bessel–Gaussian beam. The most used method of generating the Bessel–Gaussian beam is by illuminating a conically shaped element called an axicon with a Gaussian beam.^21^

Here, we demonstrate an imaging flow cytometer and cell sorter with an ultra-long depth of focus, accomplished by a scanning Bessel–Gaussian laser beam. The two-dimensional cell images can be reconstructed from one-dimensional waveform information collected from a photomultiplier tube (PMT). From this waveform, a number of cellular morphological features are quantified, and these values can be used to create appropriate gates for cell sorting. Sorting is accomplished via an integrated piezoelectric (PZT) actuator as previously described.^15,16,22^ The PZT-integrated microfluidic device is made of a cyclo-olefin copolymer (COC) material integrated with a cartridge that contains microfluidic channels and interfaces with the fluidic pumps. Both the microfluidic chip and the cartridge are injection molded and can be disposed to eliminate concerns of cross contamination.

Experiments were conducted to evaluate the sorting performance of the system for multiple sizes of polystyrene beads, label-free identification and sorting of acute myeloid leukemia (AML) cells from white blood cells, and the label-free sorting of *Scenedesmus sp.*, a green alga, from field-collected micro-organisms. Our results indicate a sorting accuracy of 97%, 97%, and 98%, respectively. We also demonstrate an increased percentage of in-focus cell images from 30% to 40% for a Gaussian beam system to >85% by using a Bessel–Gaussian beam, effectively increasing the throughput by about three folds to around 300 cells/s, limited by the response of the on-chip piezoelectric actuator and the presence of cell doublets.

## II. PRINCIPLE AND METHODS

### A. Design of the imaging system

**FIG. 1.**
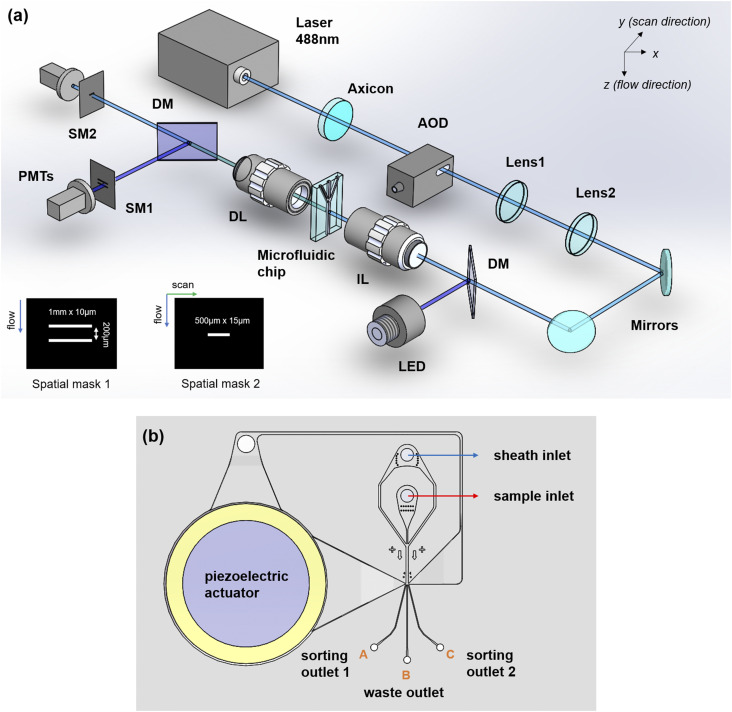
(a) Optical imaging system design. AOD, acousto-optic deflector; DM, dichroic mirror; IL, 10×/0.28 illumination objective lens; DL, 10×/0.28 detection objective lens; PMTs, photomultiplier tubes; and SM: spatial filter. The spatial masks for cell speed detection and transmission imaging are shown on the bottom left. (b) Microfluidic chip design. The chip is made of a cyclic olefin copolymer (COC) by injection molding.

The optical system design is shown in Fig. 1(a). The Gaussian beam output from a 488 nm diode laser illuminates on an axicon (AX1025-A, Thorlabs) with an angle of 0.5°. A Bessel–Gaussian beam is formed by the superposition of two sets of plane waves propagating with a cone angle. The Bessel–Gaussian beam is then modulated by using an acousto-optic deflector (OAD948, Isomet). The acoustic transducer deflects the beam to different angles along the y (scanning) direction [Fig. 1(a)] at a frequency of 200 kHz. Lens 1 performs a Fourier transform of the zero order Bessel–Gaussian beam to create an annulus-shaped beam at its focal plane. This annulus-shaped beam is then magnified by lens 2 before reaching the exit pupil of a 10× illumination objective lens (378-803-3, Mitutoyo). The illumination objective lens transforms the annulus-shaped beam back to a Bessel–Gaussian beam onto the cells in the microfluidic channel. The position of the AOD is conjugated with the back focal plane of the objective lens. This schematic creates a fan scan of the laser beam at the front focal plane. The microfluidic chip, which is made of a cyclic olefin copolymer (COC) as shown in Fig. 1(b), is put at the front focal plane.

Our design uses a single PMT detector and an AOD-scanned CW laser to encode the 2D cell transmission profile into a temporal signal, which can be used as the gating criteria for cell sorting and classification. A spatial mask [mask 2 in Fig. 1(a)] with one 500 × 15 *µ*m^2^ slit is put at the image plane of the 488 nm laser channel, which creates a 50 × 1.5 *µ*m^2^ transparent area at the focal plane. The slit is aligned to the center of the Bessel–Gaussian beam. As a result, the sidelobes of the Bessel–Gaussian beam along the flow direction are blocked while the sidelobes along the scanning direction can pass the slit.

Since the cell speed in the microfluidic channel is position dependent and the speed information is required to correctly relate the temporal waveform to the cell image, we use a 455 nm LED, a PMT, and a spatial mask [mask 1 in Fig. 1(a)] to detect the speed of each individual cell.^15^ The spatial mask contains two 1 × 10 *µ*m^2^ slits separated in the cell flow (z) direction, placed at the image plane of a 455 nm LED channel. The speed of each cell is obtained by dividing the slit distance with the magnification factor (10× in our case) and the time difference between the minima in the LED transmission signal. In our experiment, cell speeds are typically between 10 and 25 cm/s with an average speed of around 20 cm/s.

The microfluidic sorting chip was made of COC (Cyclic Olefin Copolymer) by injection molding. COC was chosen due to its high transparency in the visible wavelength, low autofluorescence, and low fabrication cost. The piezoelectric actuator was attached to the top of the COC microchip via a thin layer of double-sided PSA (pressure sensitive adhesive). The sample stream is focused by the sheath flow hydrodynamically. When a target cell is detected, the piezo-actuator is triggered to push or pull the target cell to sorting outlet 1 or 2 and eventually into either the collection tubes or specific wells in a 384-well plate. Cells that are not of interest travel through the center channel to the waste outlet.

### B. Simulation of the Bessel–Gaussian beam transmission signal

**FIG. 2.**
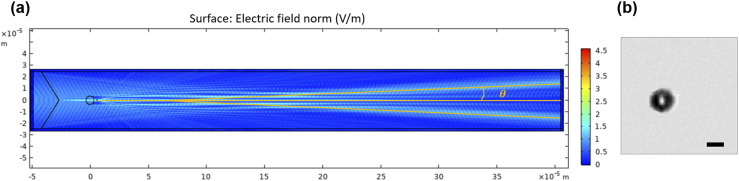
(a) COMSOL simulation of the electric field when a Bessel–Gaussian beam illuminates on the center of a 7 *µ*m bead. (b) Example transmission image of a 7 *µ*m bead generated by the image-guided cell sorter using a scanning Bessel–Gaussian beam. The image was reconstructed using the mathematical algorithm discussed in Sec. II D. Scale bar: 5 *µ*m.

To gain insight into the transmission of a Bessel–Gaussian beam through an object, we use the COMSOL Multiphysics simulation software to show how a 7 *µ*m bead (n = 1.6) changes the optical intensity distribution of a Bessel–Gaussian beam (Fig. 2). Since our system measures the far field of the transmitted light, we simulate the electric field distribution 400 *µ*m away from the bead to satisfy the Fraunhofer far-field condition. When there is no object in the interrogation zone, the laser light transmits through the slit and generates a constant DC background. When the laser beam intersects the bead, the light will be partially reflected and partially diffracted. If the diffraction angle *θ* is greater than the collection angle of the detection objective lens, the light intensity on the PMT decreases, resulting in a dark region in the transmission image of the 7 *µ*m bead due to the combined effects of reflection and diffraction assuming the effect of light absorption is negligible. According to the simulation, when the Bessel–Gaussian beam hits the center of the 7 *µ*m bead, the calculated diffraction angle *θ* is around 2°, much smaller than the collection angle of the detection objective (10×, NA = 0.28). Thus, the small angle diffraction beam can pass the slit and reach the PMT, producing a “bright spot” at the center of the image of the bead. This explains why we observe a bright spot at the center of the restored bead image from the transmitted signal [Fig. 2(b)]. As a general rule, areas of large optical density and large angle scattering give rise to dark regions; areas of low optical density and small angle scattering give rise to bright regions in the restored transmission images.

### C. Depth of focus comparison

**FIG. 3.**
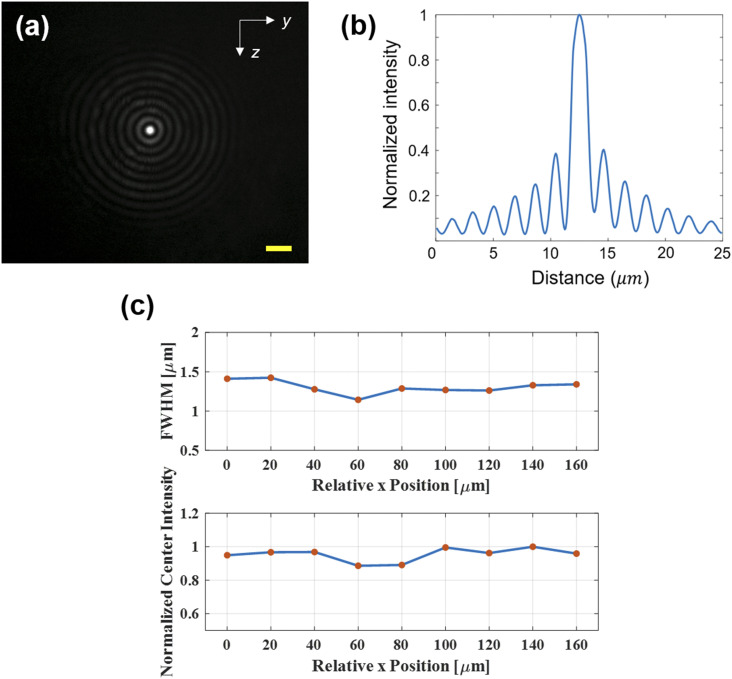
Bessel–Gaussian beam profile. (a) Camera measured beam profile at the image plane. (b) Normalized intensity from the center of the Bessel–Gaussian beam. (c) Full-width-at-half-maximum and normalized light intensity of the main lobe of the Bessel–Gaussian beam.

As discussed previously, the main motive of using a Bessel–Gaussian beam to replace a Gaussian beam is to extend the focal depth such that objects in different positions in a microfluidic channel and different cross sections of the cell can all be focused to generate high fidelity 2D cell image information. Figure 3 shows the intensity profile and focal depth of the Bessel–Gaussian beam measured by a camera. Figures 3(a) and 3(b) show the intensity profile of the Bessel–Gaussian beam at the image plane. The full width at half maximum (FWHM) of the center lobe is between 1 and 1.5 *µ*m. As expected, a significant amount of energy is in the sidelobes, which excite areas outside the central spot and complicate the waveform analysis when we use a single PMT for detection to keep the system simple and at low cost. A mathematical algorithm to be discussed in Sec. II D is required to deconvolve the signal when we reconstruct the transmission image. To measure the focal depth, beam profiles at different depths are recorded by moving the detection objective lens along the beam propagation (x) direction. Both the maximum intensity and FWHM of the center lobe have relatively small changes within a distance of 160 *µ*m, as shown in Fig. 3(c). In contrast, a Gaussian beam produced by the same objective lens has a much shorter focal depth of about 7.37 *µ*m.

To assess how the extended focal depth of a Bessel–Gaussian beam can improve the detection yield compared to a Gaussian beam, we ran a mixture of cells and beads, including 15 *µ*m beads, 7 *µ*m beads, HEK 293T cells, MCF7 cells, and Hela cells, in both Gaussian beam and Bessel–Gaussian beam image-guided cell sorters. The results are summarized in Table I. In the Gaussian beam system, the short focal depth cannot keep the majority of objects in focus due to the wide distribution of the objects along the microfluidic channel. Except for 15 *µ*m beads that tend to take a stable position in the channel, only 30%–40% 7 *µ*m beads, and only 40%–60% cells are in focus. In sharp contrast, >90% of 7 *µ*m beads, 98% of 15 *µ*m beads, and 85% of the cells of all kinds are in focus in the Bessel–Gaussian beam system.

**TABLE I.** Comparison of the ratio of in-focus objects between the scanning Gaussian beam system and the scanning Bessel–Gaussian beam system.

	Gaussian system	Bessel–Gaussian system
7 *µ*m beads	30%–40%	90%–95%
15 *µ*m beads	∼98%	∼98%
Cell mixture	40%–60%	85%–90%

**FIG. 4.**
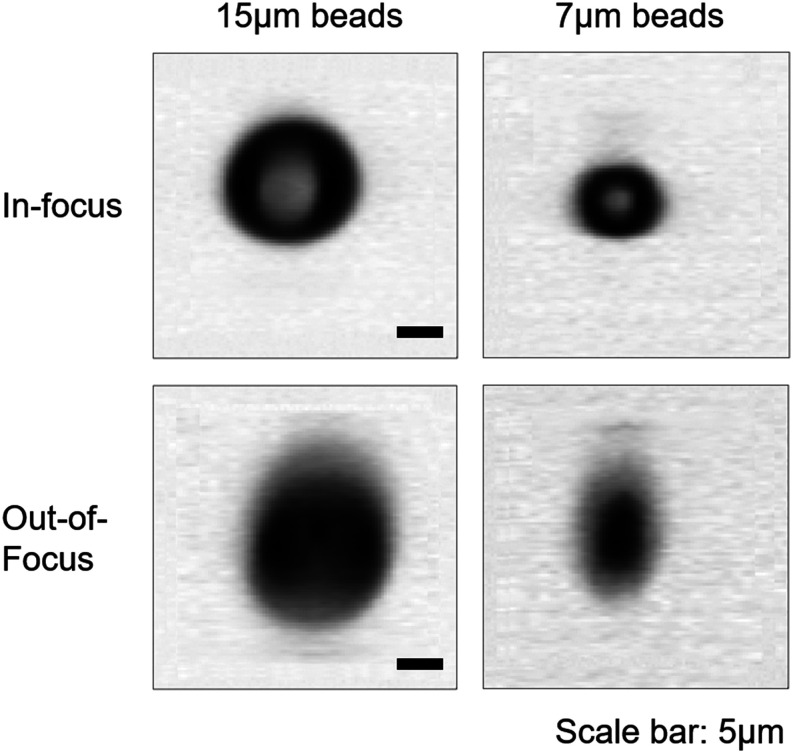
Examples of in-focus (first row) and out-of-focus (second row) images for 15 and 7 *µ*m beads, generated by a scanning Gaussian beam image-guided cell sorter. In comparison, nearly all images from the scanning Bessel–Gaussian beam system are well in focus (see Fig. 5). Scale bar: 5 *µ*m.

Figure 4 shows example in-focus and out-of-focus 15 *µ*m bead and 7 *µ*m bead images generated by the Gaussian beam system. In sharp contrast, the vast majority of both 15 and 7 *µ*m diameter beads are well focused for the Bessel–Gaussian beam system.

### D. Image reconstruction algorithm

In this section, we describe the mathematical algorithm to reconstruct images from the label-free, transmission signal by a Bessel–Gaussian beam. It is noted that because of the correlation between the PMT temporal signal and the image features, we do not need to use the restored cell images as gates to sort cells. Instead, we sort cells directly from the features in the waveform, thus saving time and resources for real time signal processing. Therefore, image reconstruction can be performed off-line for validation of the results and improved human–machine interface when users would like to observe image differences between sorted and unsorted cells and visualize image related features, such as size, shape, granularity, and contrast.

The electric field of a Bessel–Gaussian beam can be written as

$$E_{BG}\left(r,x\right)=E_{0}J_{0}\left(k_{r}r\right)\frac{w_{0}}{w\left(x\right)}e^{-\frac{r^{2}}{w_{0}^{2}}}\cdot e^{-ik_{x}x}e^{-i\emptyset },$$ (1)

where $r=\sqrt{y^{2}+z^{2}}$ is the distance from the center of the Bessel–Gaussian beam. *E*_0_ is a field amplitude constant. *k*_*r*_ is the wavevector in the transverse plane and $k_{r}^{2}+k_{x}^{2}=k^{2}$. *w*_0_ is the waist width of the Gaussian amplitude. $\emptyset =tan^{-1}\frac{x}{x_{0}}$. *x*_0_ is the Rayleigh length of the Gaussian beam.

We use *n*(*x*, *y*, *z*) to denote the cell or bead index profile $n\left(x,y,z\right)=n_{o}+\Delta n\left(x,y,z\right)$. We assume *n*_*o*_ is the index of water and Δ*n* > 0 since the index of cells and beads is greater than the index of water. Assume the cell or bead thickness is within *x*_*c*_. For the 2D imaging system, we cannot resolve the index change along the beam propagation direction, so we make the following approximation:

$$\int_{0}^{x_{c}}\Delta n\left(x,y,z\right)dx=\Delta \bar{n}\left(y,z\right)x_{c}.$$ (2)

Adding a slit in parallel with the laser scanning (y) direction on the image plane and assuming the slit is narrow enough to be approximated by a 1D delta function in its transmission characteristic, the transmitted field focused by a lens and after the slit can be approximated by (3),

$$\begin{array}{ll} E_{t}\left(y^{\prime },z^{\prime }\right) & =E_{0}^{\prime }e^{-ik_{o}x}e^{-i\emptyset }\frac{w_{0}}{w\left(x\right)}\int_{y}\int_{z}\delta \left(z-z^{\prime }\right)\\ & \;\times \left(\frac{2\sqrt{n_{o}(n_{o}+\Delta \bar{n}\left(y,z\right)}}{2n_{o}+\Delta \bar{n}\left(y,z\right)}\right)\\ & \;\times J_{0}\left[k_{r}\sqrt{{\left(y^{\prime }-y\right)}^{2}+{\left(z^{\prime }-z\right)}^{2}}\right]e^{-\frac{{\left(y^{\prime }-y\right)}^{2}+{\left(z^{\prime }-z\right)}^{2}}{w_{0}^{2}}}\\ & \;\times e^{-ik_{o}\Delta \bar{n}\left(y,z\right)x_{c}}dydz. \end{array}$$ (3)

The term $\left(\frac{2\sqrt{n_{o}(n_{o}+\Delta \bar{n}\left(y,z\right)}}{2n_{o}+\Delta \bar{n}\left(y,z\right)}\right)$ in Eq. (3) is the approximate transmission coefficient assuming there is no absorption. Here, (*y*, *z*) refers to the transverse coordinate in the object plane, and (*y*′, *z*′) refers to the transverse coordinate in the image (detection) plane. For simplicity, we have transformed the actual position (*Y*′, *Z*′) in the image plane into (*y*′, *z*′) by defining $y^{\prime }=\frac{Y^{\prime }}{M}$ and $z^{\prime }=\frac{Z^{\prime }}{M}$ with *M* being the magnification of the detection optics.

Also note that (*y*′, *z*′) is related to the time by the following relations:

$$y^{\prime }=\frac{FOV_{y}}{T}\;t,$$ (4a)

$$z^{\prime }=v_{flow}t,$$ (4b)

where *FOV*_*y*_ is the field-of-view in the y (scanning) direction, T is the time for each AOD scan (5 *μ*s in our system), and *v*_*flow*_ is the flow speed of the object (around 20 cm/s in our system). For a 40 *μ*m field-of-view in the scanning direction and an AOD scanning period of 5 *μ*s, the scanning speed is 8 m/s, which is 40 times faster than the average cell travel speed. This allows us to treat the scanning along the y axis as if the cell is nearly still in the z axis.

From the relations in (4), we can relate a signal in the time domain to the space domain, thus reconstructing the image from a temporal waveform.

To analyze the detected cell transmission signal behind the slit when the center of the scanning Bessel–Gaussian beam is at a given position in the flow (*z*′) direction, we can represent the transmitted field in (5) under a given position *z*′,

$$\begin{array}{ll} E_{t}\left(y^{\prime }\right){|}_{z^{\prime }} & \propto \int_{y}\left(\frac{2\sqrt{n_{o}(n_{o}+\Delta \bar{n}\left(y\right){|}_{z^{\prime }}}}{2n_{o}+\Delta \bar{n}\left(y\right){|}_{z^{\prime }}}\right)J_{0}\left[k_{r}\sqrt{{\left(y^{\prime }-y\right)}^{2}}\right]\\ & \;\times e^{-\frac{{\left(y^{\prime }-y\right)}^{2}}{w_{0}^{2}}}e^{-ik_{o}\Delta \bar{n}\left(y\right){|}_{z^{\prime }}x_{c}}dy. \end{array}$$ (5)

Equation (5) shows that $E_{t}\left(y^{\prime }\right){|}_{z^{\prime }}$ is the convolution of the index function $\left(\frac{2\sqrt{n_{o}(n_{o}+\Delta \bar{n}\left(y\right){|}_{z^{\prime }}}}{2n_{o}+\Delta \bar{n}\left(y\right){|}_{z^{\prime }}}\right)e^{-ik_{o}\Delta \bar{n}\left(y\right){|}_{z^{\prime }}x_{c}}$ and the Bessel–Gaussian function $J_{0}\left[k_{r}y\right]e^{-\frac{y^{2}}{w_{0}^{2}}}$ along the scanning (y) direction. To save computational power for image reconstruction, we approximate the Bessel function $J_{0}\left[k_{r}y\right]$ by a series of delta functions at its maxima and minima,

$$J_{o}\left(u\right)∼\underset{m}{\sum }c_{\max,m}\delta \left(u-u_{\max,m}\right)+\underset{n}{\sum }c_{\min,n}\delta \left(u-u_{\min,n}\right).$$ (6)

*u*_max,*m*_: positions of the *m*th maximum of $J_{o}\left(u\right)$. $J_{o}\left(u_{\max,m}\right)>0$; *m* = 0, ±1, ±2, ±3, ….
*u*_min,*n*_: positions of the *n*th minimum of $J_{o}\left(u\right)$. $J_{o}\left(u_{\min,n}\right)<0$; *n* = ±1, ±2, ±3, ….

The coefficients for each delta function are defined as

$$c_{\max,m}=J_{0}\left(u_{\max,m}\right)\;m=0,\;\pm 1,\;\pm 2,\;\pm 3,\ldots ,$$

$$c_{\min,n}=J_{0}\left(u_{\min,n}\right)\;n=\pm 1,\;\pm 2,\;\pm 3,\ldots .$$

Substituting (6) into (5) and dropping the parameter *z*′ for simplicity, we obtain the following approximate expression of the transmitted E-field behind the slit:

$$\begin{array}{ll} E_{t}\left(y^{\prime }\right){|}_{z^{\prime }} & ∼\left[\sum_{m}C_{\max,m}\exp\left[-\frac{u_{\max,m}^{2}}{{\left(k_{r}w_{o}\right)}^{2}}\right]\left(\frac{2\sqrt{n_{o}(n_{o}+\Delta \bar{n}\left(y^{\prime }-\frac{u_{\max,m}}{k_{r}}\right){|}_{z^{\prime }}}}{2n_{o}+\Delta \bar{n}\left(y^{\prime }-\frac{u_{\max,m}}{k_{r}}\right){|}_{z^{\prime }}}\right)e^{-ik_{o}\Delta \bar{n}\left(y^{\prime }-\frac{u_{\max,m}}{k_{r}}\right){|}_{z^{\prime }}x_{c}}\right]\\ & \;-\left[\sum_{n}C_{\min,n}\exp\left[-\frac{u_{\min,n}^{2}}{{\left(k_{r}w_{o}\right)}^{2}}\right]\left(\frac{2\sqrt{n_{o}(n_{o}+\Delta \bar{n}\left(y^{\prime }-\frac{u_{\min,n}}{k_{r}}\right){|}_{z^{\prime }}}}{2n_{o}+\Delta \bar{n}\left(y^{\prime }-\frac{u_{\min,n}}{k_{r}}\right){|}_{z^{\prime }}}\right)e^{-ik_{o}\Delta \bar{n}\left(y^{\prime }-\frac{u_{\min,n}}{k_{r}}\right){|}_{z^{\prime }}x_{c}}\right]. \end{array}$$ (7)

Representing $E_{t}\left(y^{\prime }\right)∼A-B$ in brief form, we can write the transmitted power through the slit as

$$J_{t}\left(y^{\prime }\right){|}_{z^{\prime }}\propto E_{t}^{*}\left(y^{\prime }\right){|}_{z^{\prime }}E_{t}\left(y^{\prime }\right){|}_{z^{\prime }}\propto AA^{*}+BB^{*}-AB^{*}-BA^{*}.$$ (8)

It can be shown that (8) can be approximated as

$$\begin{array}{ll} J_{t}\left(y^{\prime },x\right) & ∼\frac{4n_{o}(n_{o}+\Delta \bar{n}\left(y^{\prime }\right){|}_{z^{\prime }}}{{\left(2n_{o}+\Delta \bar{n}\left(y^{\prime }\right){|}_{z^{\prime }}\right)}^{2}}+\left[\underset{m\neq 0}{\sum }C_{\max,m}^{2}\exp\left[-\frac{2u_{\max,m}^{2}}{{\left(k_{r}w_{o}\right)}^{2}}\right]\frac{4n_{o}(n_{o}+\Delta \bar{n}\left(y^{\prime }-\frac{u_{\max,m}}{k_{r}}\right){|}_{z^{\prime }}}{{\left(2n_{o}+\Delta \bar{n}\left(y^{\prime }-\frac{u_{\max,m}}{k_{r}}\right){|}_{z^{\prime }}\right)}^{2}}\right]\\ & \;+\left[\underset{n}{\sum }C_{\min,n}^{2}\exp\left[-\frac{2u_{\min,n}^{2}}{{\left(k_{r}w_{o}\right)}^{2}}\right]\frac{4n_{o}(n_{o}+\Delta \bar{n}\left(y^{\prime }-\frac{u_{\min,n}}{k_{r}}\right){|}_{z^{\prime }}}{{\left(2n_{o}+\Delta \bar{n}\left(y^{\prime }-\frac{u_{\min,n}}{k_{r}}\right){|}_{z^{\prime }}\right)}^{2}}\right]. \end{array}$$ (9)

To obtain (9), we have ignored summations of terms with a random phase *φ*_*mn*_, such as $\sum_{m,n}e^{-i\varphi_{mn}}$, where $\varphi_{m,n}=k_{o}x_{c}\left[\Delta \bar{n}\left(y^{\prime }-\frac{u_{\max,m}}{k_{r}}\right){|}_{z^{\prime }}-\Delta \bar{n}\left(y^{\prime }-\frac{u_{\min,n}}{k_{r}}\right){|}_{z^{\prime }}\right]$. In other words, we have only kept the phase-matched terms with *φ*_*mn*_ = 0.

According to (9), when the beam center hits a high index spot, $\frac{4n_{o}(n_{o}+\Delta \bar{n}\left(y^{\prime }\right)}{{\left(2n_{o}+\Delta \bar{n}\left(y^{\prime }\right)\right)}^{2}}{|}_{z^{\prime }}<1$, the light intensity through the slit would yield a lower than 100% transmission. When the beam center hits a low index position (e.g., water only), the first term in (9) is maximum, but the values in the second and third terms depend on the index values $\Delta \bar{n}\left(y^{\prime }-\frac{u_{\max,m}}{k_{r}}\right){|}_{z^{\prime }}$ and $\Delta \bar{n}\left(y^{\prime }-\frac{u_{\min,n}}{k_{r}}\right){|}_{z^{\prime }}$ relative to the maxima and minima of the sidelobes. In order to reconstruct the image from the measured PMT signal, we need to solve $\Delta \bar{n}\left(y\right){|}_{z}$ or simply $\Delta \bar{n}\left(y,z\right)$. Next, we describe the algorithm to obtain $\Delta \bar{n}\left(y,z\right)$ from Eq. (9).

Define $f\left(y,z\right)=\frac{4n_{o}(n_{o}+\Delta \bar{n}\left(y\right)}{{\left(2n_{o}+\Delta \bar{n}\left(y\right)\right)}^{2}}{|}_{z}$. By solving $f\left(y,z\right)$, we can know the index profile of the object $\Delta \bar{n}\left(y,z\right)$.

Equation (9) can be represented in the matrix form as

$$J_{t}\left[y_{1}^{\prime },y_{2}^{\prime },\ldots ,y_{N}^{\prime };z_{j}^{\prime }\right]=\left[T\right]*f\left[y_{1}^{\prime },y_{2}^{\prime },\ldots ,y_{N}^{\prime };z_{j}^{\prime }\right],$$ (10)

where *j* denotes the z position of the cell in the flow direction after *j* times of AOD scans. The T-matrix is a 251 × 251 matrix. The dimension of the matrix is determined as follows: at a sampling rate of 25 MS/s and for a single scan of 5 *µ*s, we produce 125 data points corresponding to the center positions of the Bessel–Gaussian beam over the 40 *µ*m scanning range. However, the Bessel–Gaussian beam has sidelobes. Assuming that the sidelobes on each side of the beam center span 20 *µ*m, we have the scanning Bessel–Gaussian beam cover a total range of 80 *µ*m, thus producing a total of 251 points in the transfer matrix in (10). The elements of the T-matrix are defined as follows:

$$T_{ij}=1,\;if\;i=j,\;-65⩽i,j⩽185.$$

$T_{ij}=C_{l}^{2}\exp\left[-\frac{2u_{l}^{2}}{{\left(k_{r}w_{o}\right)}^{2}}\right]\equiv a_{l}^{2}$, where *u*_*l*_ is the *l*th min or max for $J_{0}\left(u\right)$ if $y_{i}^{\prime }-\frac{u_{l}}{k_{r}}=y_{j}^{\prime }$,

$$T_{ij}=0,\;otherwise.$$ (11)

Then, $\Delta \bar{n}\left(y,z\right)$ can be obtained from Eq. (12),

$$f\left[y_{1}^{\prime },y_{2}^{\prime },\ldots ,y_{N}^{\prime };z_{j}^{\prime }\right]={\left[T\right]}^{-1}*J_{t}\left[y_{1}^{\prime },y_{2}^{\prime },\ldots ,y_{N}^{\prime };z_{j}^{\prime }\right].$$ (12)

From Eq. (12), we can reconstruct the transmission image of the object from the PMT signal. More detailed analyses can be found in the supplementary material.

### E. Waveform-based real-time sorting

The mathematical algorithm in Sec. II D can recover the object image from the PMT signal. However, the computation of 251 × 251 matrix multiplication is time-consuming and can limit the throughput. On the other hand, because most cell features, including size, spottiness, and granularity, are encoded in the PMT output waveform, we can extract many image features that differentiate cell types directly from the temporal waveform without reconstructing the 2D cell images. This saves tremendous computation time and resources, and the method is suitable for cell sorting by image features. For all sorting experiments reported in this paper, we define gating based on the characteristics of the temporal waveform, which are closely correlated with specific image features. We then use the mathematical algorithm discussed in Sec. II D to reconstruct the cell transmission images off-line for verification purposes. To quantify the sorting accuracy, we also apply additional methods, such as staining and microscopy, to verify the performance of waveform-based image-guided cell sorting.

**FIG. 5.**
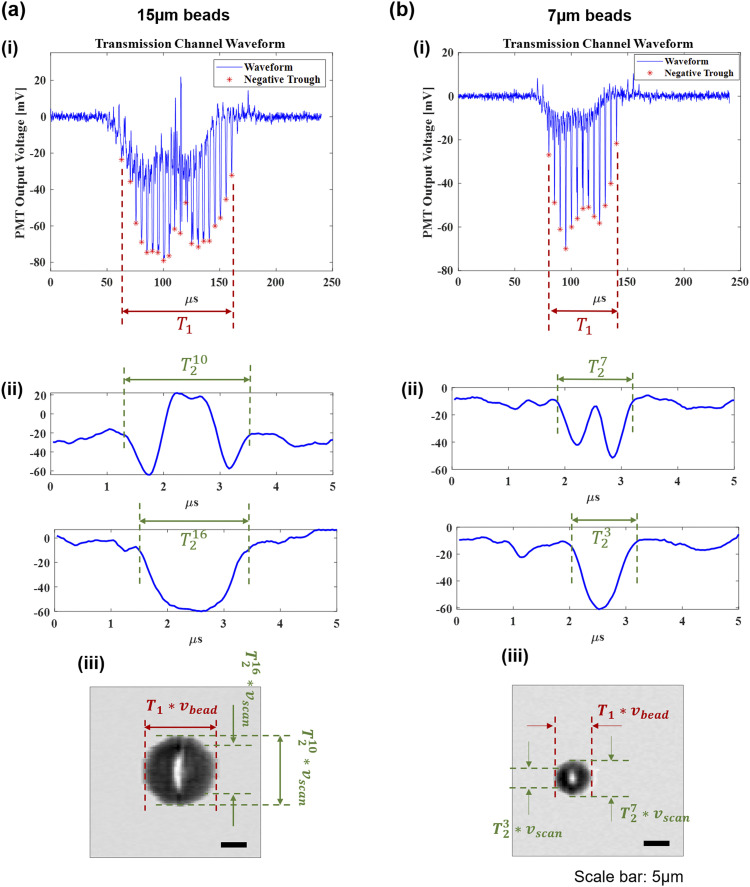
Transmission PMT signals for 15 and 7 *µ*m beads and reconstructed images. Scale bar: 5 *µ*m. (i) The overall signals. Each “*” represents the peak of each scan. The product of the bead speed *v*_*bead*_ and the width of the overall envelope *T*_1_ produces the bead dimension along the flow direction. (ii) Detailed waveforms for a single 5 *µ*s AOD scan. At each specific z position, the dimension of the bead along the scanning direction is *T*_2_ ∗ *v*_*scan*_, where *v*_*scan*_ = 8 m/s is the beam scanning speed. (iii) Reconstructed transmission images of a 15 and 7 *µ*m bead. The relations between the temporal waveforms and the image features are also indicated in the figures.

Figure 5 shows an example of how the temporal waveform carries features about particle size and how we can use the waveform features to distinguish 15 and 7 *µ*m diameter beads. When there is no object in the microfluidic channel, the scanning Bessel–Gaussian beam transmits through the slit and the PMT shows a periodic background signal, caused by any imperfections or dust particles in the COC microfluidic chip intersected by the laser beam. Since these features are still, they appear to be periodic in each scan and can be subtracted by software. When a cell or bead travels through the optical interrogation area, it creates an instantaneous change in the PMT output signal on top of the background. The PMT waveforms in Figs. 5(a-i) and 5(b-i) show an envelope with a series of spikes. Each spike represents a single scan spanning a duration of 5 *µ*s, and the width of the spike is proportional to the size of the bead along the scanning (y) direction. On the other hand, the width of the overall signal envelope is proportional to the dimension of the bead in the flow direction after correction of the effect of the flow speed. Based on this argument, we develop the following sorting criterion that is equivalent to the particle size.

We find the time interval between the first negative peak and the last negative peak *T*_1_, which corresponds to the duration when the bead crosses the optical interrogation zone defined by the width of the slit in the spatial mask. The bead length *L* along the flow direction equals *T*_1_ ∗ *v*_*bead*_, where *v*_*bead*_ is the bead traveling speed. We then analyze the detailed waveform of each 5 *µ*s scan (labeled by “*” in the envelope waveform) to find the bead dimension in the scanning direction. Figures 5(a-ii) and 5(b-ii) show the detailed waveform of each 5 *µ*s scan at a given z position. By slicing the object into N sections along the z position, the object width at the *n*th section can be represented as $T_{2}^{n}*v_{scan}$ with *n* being the index of the z position and *v*_*scan*_ is the beam scanning speed (*v*_*scan*_ = 8 m/s). Figure 5(a-ii) shows two (10th and 16th) of such scans for a 15 *µ*m bead. The tenth scan gives the largest value of $T_{2}^{n}*v_{scan}$, indicating the widest part (i.e., diameter) of the bead. Similar characteristics can be found in the waveform of 7 *µ*m beads. The above example demonstrates how one can relate the temporal waveform features to the geometric features of a traveling object, such as size, shape, and aspect ratio.

## III. EXPERIMENTAL RESULTS

### A. Sorting of 10 and 15 *µ*m beads

**FIG. 6.**
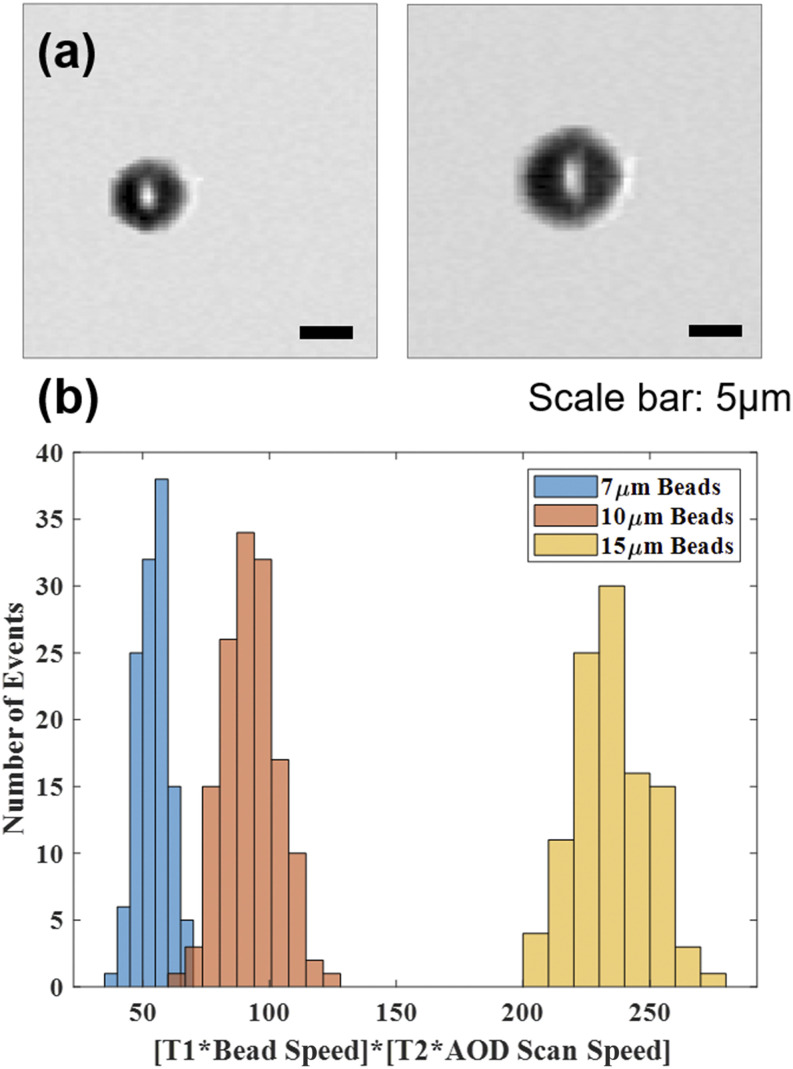
Images and histograms of polystyrene beads generated by the Bessel–Gaussian beam image-guided cell sorter. (a) Transmission images of polystyrene beads with 7 *µ*m (left) and 10 *µ*m (right) diameter. Scale bar: 5 *µ*m. (b) Histogram of (*T*_1_ ∗ *v*_*bead*_) ∗ (*T*_2_ ∗ *v*_*scan*_) for 7, 10, and 15 *µ*m beads.

To validate the sorting algorithm described above, a sorting experiment was done using 7, 10, and 15 *µ*m beads. The histogram of (*T*_1_ ∗ *v*_*bead*_) ∗ (*T*_2_ ∗ *v*_*scan*_) is shown in Fig. 6(b). To evaluate the sorting performance, we sorted 10 *µ*m beads from a 1:1 mixture of 7 and 10 *µ*m beads, as well as 15 *µ*m beads from a 1:1 mixture of 7 and 15 *µ*m beads. The sorted beads were imaged using a microscope to verify the sorting accuracy. The first experiment demonstrated a sorting purity of 97%, verified by 233 microscope images; the second experiment demonstrated 100% sorting purity, verified by 173 microscope images.

### B. Label-free sorting of leukemia cells

Blood cancers, such as acute myeloid leukemia (AML), are estimated to account for 9.9% of the 1.8 × 10^6^ new cancer cases diagnosed in 2020.^23^ Leukemia, lymphoma, and myeloma are expected to account for 9.4% of all cancer deaths in 2020.^24^

Acute myeloid leukemia is derived from the myeloid line of blood cells and is characterized by its rapid and unchecked growth of abnormal cells in the bone marrow that interferes with normal blood cell production. Diagnosis usually occurs via bone marrow aspiration or antibody-specific blood tests.^25^ However, these require costly panels and tedious procedures. An image-guided cell sorter enables the identification and subsequent sorting of AML cells without any antibody or fluorescent labeling, aiding early detection and eliminating the need for costly reagents and tedious laboratory procedures.

**FIG. 7.**
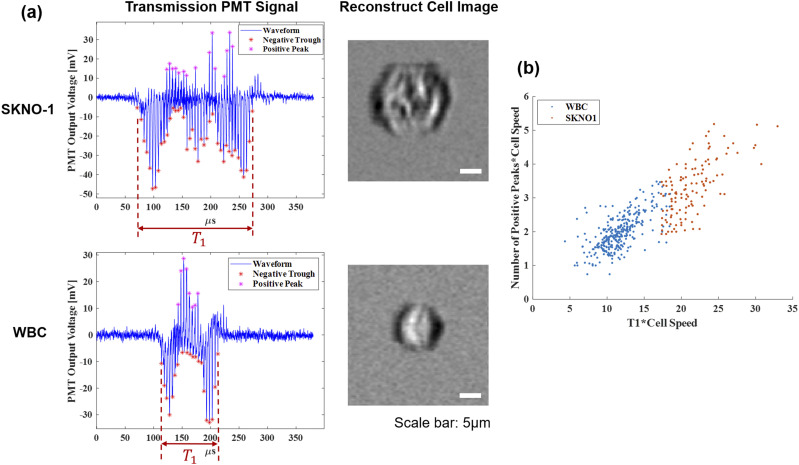
(a) Transmission PMT signals and images for SKNO-1 and WBC generated by the Bessel–Gaussian beam image-guided cell sorter. Scale bar: 5 *µ*m. (b) Distribution plots using these two parameters: *T*_1_ ∗ *v*_*cell*_ and *N* ∗ *v*_*cell*_, where *N* is the number of positive peaks in the PMT waveform. Multiplication of cell speed to both parameters removes feature distortions due to cell speed variations.

In a proof-of-concept experiment, patient-derived SKNO1 acute myeloid leukemia (AML) cells were cultured in cell culture media (90% RPMI + 8% FBS + 1% penicillin + 1% streptomycin) at 37 °C with 5% CO_2_. The SKNO1 cells were spiked into white blood cells from healthy donors (San Diego Blood Bank, 3636 Gateway Center Ave. Suite 100, San Diego). A number of feature parameters were extracted from the transmission waveform of these cells, which are intuitively related to cell area, perimeter, granularity, roughness, contrast, and texture. The most distinguishing features between SKNO1 cells and white blood cells were determined to be *T*_1_ ∗ *v*_*cell*_ and the number of positive peaks of the waveform. The former is related to the cell size and the latter to the intracellular granularity. To demonstrate image-guided label-free cell sorting, a 2D plot of these parameters was generated and the appropriate gating parameters were chosen to sort SKNO1 cells from healthy white blood cells in a ratio of 1:50 (Figs. 7). To evaluate the cell sorting, Wright–Giemsa staining was performed. The full details for the staining procedure can be found in the supplementary material. The sorted cells were collected in a tube and deposited on a polyester transparent membrane filter (1300019, Sterlitech). Wright–Giemsa staining was performed, and the stained cells were imaged using bright-field microscopy. A total of 124 SKNO1 cells were imaged from a total of 128 cells found on the membrane, giving rise to a sorting purity of 97%. Given the initial population of 2% SKNO1 cells, the sorting has enriched the sample by 1600 times.

**FIG. 8.**
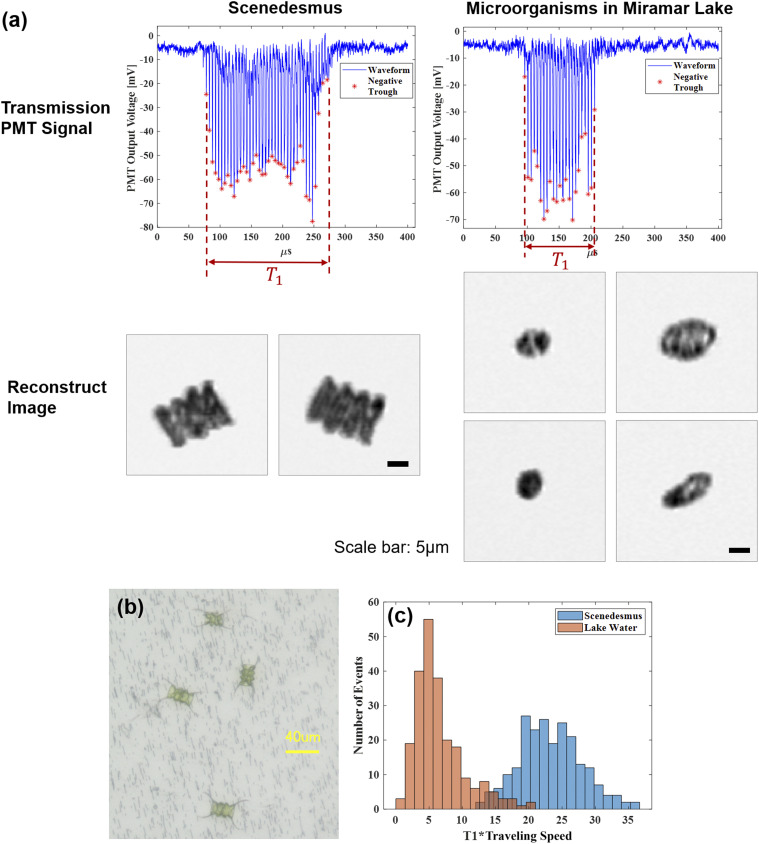
(a) Transmission channel waveforms and reconstructed images of *Scenedesmus* and micro-organisms in Miramar Lake water. Scale bar: 5 *µ*m. (b) Optical microscope images of *Scenedesmus* being sorted on membrane filter. (c) Histogram of *T*_1_ ∗ *v*_*algae*_ for *Scenedesmus* and other micro-organisms in lake water.

### C. Label-free sorting of *Scenedesmus sp.*

Algae are a group of photosynthetic, eukaryotic organisms that can be found in oceans, waterways, lakes, and soils all over the world. Algae are commonly used to monitor environmental changes and have a number of industrial uses, including the production of biodiesel, ceramic products, and glass products, in wastewater and oil spill cleanup, and in the biotechnology field as anticoagulant, antiviral, and antitumor agents.^26–29^ Despite their usefulness, little is known regarding the majority of these algae, with the estimated number of microalgae species exceeding 1 × 10^6^.^30^ In comparison, the best algae culture collections often contain only a few thousand species.^31^ Isolation of microalgae species from the environment is a useful and necessary approach to understanding these organisms and uncovering potential technological solutions. Traditionally, these organisms are isolated by hand using micropipettes or capillary tubes, or by fluorescence-activated cell sorting, and are subsequently cultured.^32^ However, the throughput and usefulness of these approaches are limited, as microalgae and other micro-organisms experience complex relationships with surrounding organisms that affect algae phenotype.

*Scenedesmus sp.* is one of the most common freshwater green algae. These colonial, non-motile algae have been researched for their high biomass productivity and efficiency at capturing CO_2_.^33^ *Scenedesmus* is capable of producing many types of biofuels and has been most extensively studied for biodiesel production. As there are over 70 taxonomically accepted species of *Scenedesmus*, including some with unique properties that only exist in local populations, the high-throughput identification and sorting of these algae from field-collected samples could unlock new opportunities.^33^

As a proof-of-concept sorting experiment, *Scenedesmus* (Carolina Biological Supply, 152510) were spiked into field-collected micro-organisms (Miramar Lake, San Diego, CA) in a ratio of 1:5. The sample was run through a 35 *µ*m filter to remove clumps and large particles. The distinguishing feature of *Scenedesmus* from the other micro-organisms was *T*_1_ ∗ *v*_*algae*_, which is intuitively related to size. A histogram with these parameters was generated, and the appropriate portion was gated (Figs. 8). The sorted samples were collected into tubes and visualized using bright-field microscopy. From a total of 253 sorted cells verified by a microscope, 248 of them were *Scenedesmus* and 5 were other micro-organisms, resulting in a sorting purity of 98%.

## IV. DISCUSSION AND CONCLUSION

Leveraging the unique properties of Bessel beam illumination, we present a microfluidic, label-free image-guided cell sorter with an ultra-long depth of focus, resulting in a threefold increase in the number of in-focus cells compared with Gaussian beam systems. Proof-of-concept experiments were demonstrated with high sorting purity using the label-free transmission waveform features as sorting criteria. For the sorting of polystyrene beads, SKNO1 leukemia cells, and *Scenedesmus* green algae, our results indicate a sorting purity of 97%, 97%, and 98%, respectively. Because of the sidelobes inherent to the Bessel beam, a significant amount of computation is required to restore the cell image from the measured temporal signal, and this computation time can limit the throughput. The current system keeps refreshing the cell images for data visualization with a processing time of ∼3 ms using an FPGA processor (Xilinx Kintex-7 XC7K410T). It is estimated that the image processing time can be reduced to less than 500 *μ*s by using a more powerful FPGA, such as Xilinx Virtex XCVU440.

By approximating the zero-order Bessel function by a series of delta functions at its maxima and minima, we have developed an effective mathematical algorithm to restore the cell image from the waveform. The restored images have shown sufficient quality to allow users to visualize the object that is being analyzed and sorted. More importantly, we have demonstrated the method of using the waveform features as gating criteria to sort cells with superior sorting purity, utilizing the close relations between the waveform features and the image features. The successful demonstration of this approach eliminates the need for real-time image reconstruction, greatly reducing the computation resource and time delay. On the other hand, the ability to restore the cell images from the waveform in an off-line process facilitates the human–machine interface, enhancing the operability and user-friendliness of the system.

## SUPPLEMENTARY MATERIAL

See the supplementary material for the illustration of the detailed algorithms for image reconstruction and protocols for sample preparation.

## Data Availability

The data that support the findings of this study are available from the corresponding authors upon reasonable request.
